# Delayed onset autoimmune cholangitis in a patient treated with pembrolizumab

**DOI:** 10.1093/bjrcr/uaae040

**Published:** 2024-11-01

**Authors:** Joshua Newington, Daniel Patterson, Pilar Sanchez

**Affiliations:** Addenbrooke’s Hospital, University of Cambridge, Cambridge CB2 0SP, United Kingdom; Department of Oncology, West Suffolk Hospital, Bury Saint Edmunds IP33 2QZ, United Kingdom; Department of Radiology, West Suffolk Hospital, Bury Saint Edmunds IP33 2QZ, United Kingdom

**Keywords:** pembrolizumab, autoimmune cholangitis, delayed onset

## Abstract

This case study describes a female patient in her late 70s who developed autoimmune cholangitis a year after finishing 35 cycles of pembrolizumab for the treatment of her non-small cell lung cancer. The diagnosis was initially missed and delayed; the patient’s agoraphobia and the COVID-19 pandemic were noted as contributing factors.

## Background

Pembrolizumab (Keytruda) is a humanized immunoglobin that targets programmed cell death protein 1 (PD-1) receptors on lymphocytes.^1^ The binding between PD-1 and PD-L1 prevents immune system destruction of tumour cells. It is approved for the treatment of various cancers, including epidermal growth factor receptor (EGFR) negative, anaplastic lymphoma kinase (ALK) rearrangement negative non-small cell lung cancer (NSCLC). This class of drugs, known as checkpoint inhibitors, have increasingly been used to treat and palliate a range of cancers. In recent months, pembrolizumab has been approved for use in high-risk early-stage breast cancer and in combination with platinum chemotherapies (https://www.fda.gov/drugs/resources-informationapproved-drugs/fda-approves-pembrolizumab-high-riskearly-stage-triple-negative-breast-cancer). Pembrolizumab has increased the average survival time to 26.3 months compared to 14.3 months for chemotherapy alone in NSCLC patients, and 1 in 3 patients survive more than 5 years.^2^

Despite its efficacy, pembrolizumab is not without reported side effects. Some common and relatively benign side effects include rash, pruritus, and fatigue.^3^ However, there have also been reports of severe and potentially life-threatening immune-related events such as colitis, hepatitis, nephritis, myocarditis, and pneumonitis. Onoyama et al.^4^ reported that up to 4.5% of patients receiving PD-L1 go on to develop sclerosing cholangitis, the median number of cycles in this study was 5.5.

## Case presentation

The patient, in this case, kindly provided written and informed consent for publication. A female patient in her late 70s with a past medical history of Graves’ disease with radioiodine-induced hypothyroidism presented with a non-productive cough in 2017. A CT scan revealed right hilar and right lower lobe masses with associated lymphadenopathy. Later, a biopsy of the adrenal metastases showed the T3N0M1b NSCLC had no EGFR or ALK mutations but had a 90%-100% PD-L1 expression. Treatment consisted of 35 rounds of intravenous pembrolizumab, and initial side effects included weight loss, diarrhoea, and abdominal pain. Throughout the treatment, liver function markers remained unremarkable.

Seven months after the first round, the patient experienced rheumatological symptoms, including pain in the knee joint and gout-like symptoms. Five months later, the patient complained of loose stool, which was suspected to be a consequence of immunotherapy-induced colitis; however, endoscopy showed no signs of inflammation, and this settled down quickly and did not require steroids.

**Figure 1.**
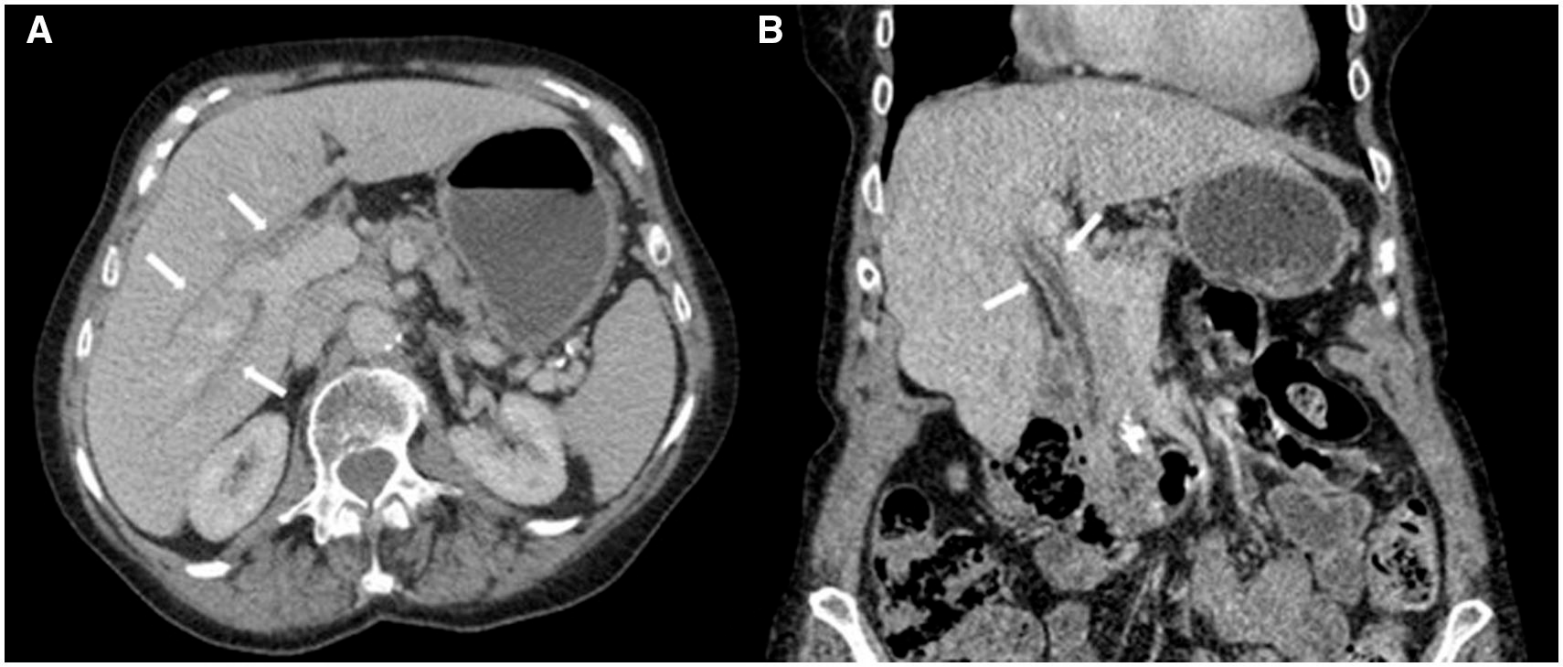
CT performed in September 2020: (A) Axial contrast-enhanced CT image showing mild intrahepatic biliary dilatation with enhancing diffuse ductal wall thickening (white arrows). (B) Coronal reformatted image showing mildly dilated common bile duct with enhancing thickened wall (white arrows).

**Figure 2.**
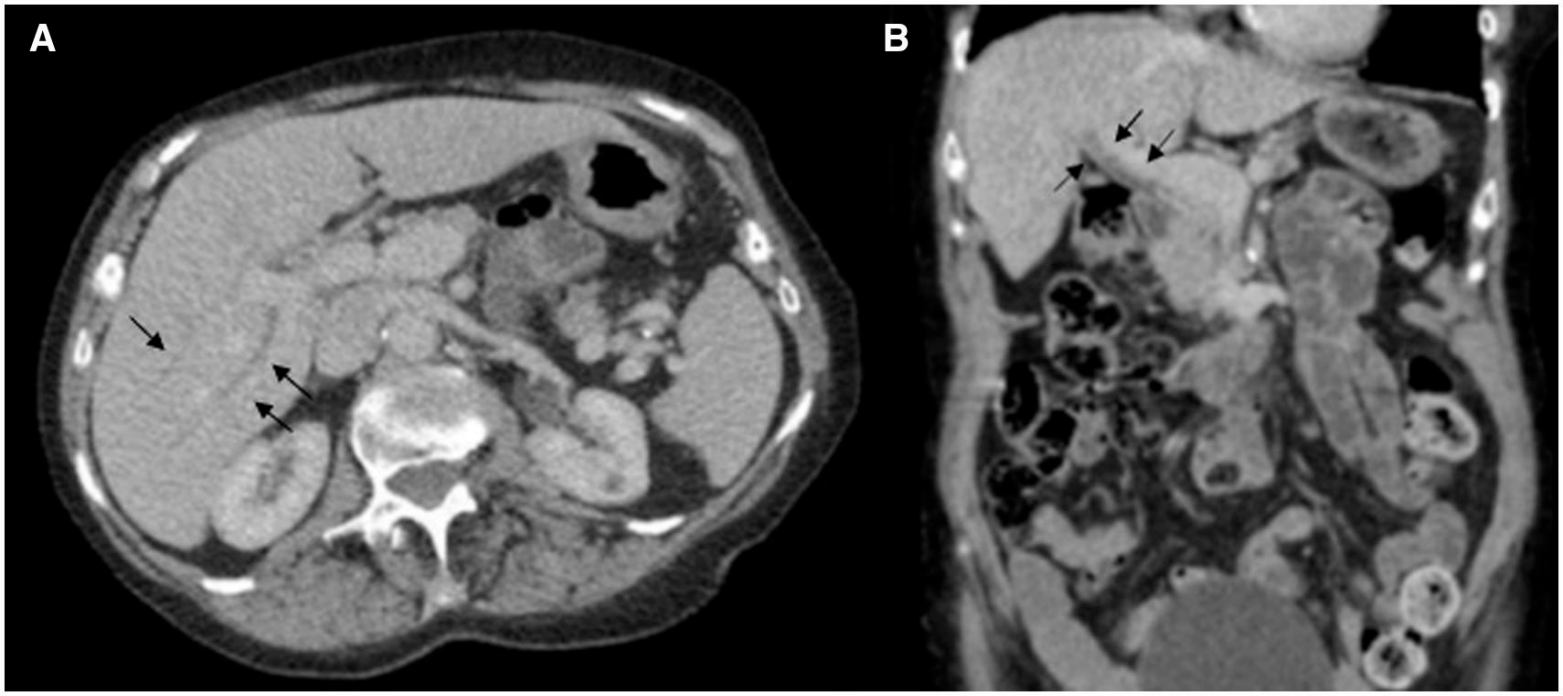
CT performed in February 2021: (A) Axial contrast-enhanced CT image showing mild intrahepatic biliary dilatation with enhancing diffuse ductal wall thickening, but less thickened than in previous study in Figure 1, concerning for cholangitis (black arrows). (B) Coronal reformatted image showing mildly dilated common hepatic and common bile duct with enhancing thickened wall (black arrows).

During 2020, the patient had 3 monthly telephone consultations and appeared clinically well apart from slight weight gain. In February 2020, the patient completed her course of pembrolizumab. In September 2020, a CT scan report described stable disease but failed to detect and report the biliary abnormalities (Figure 1). Unexpectedly, a blood test in December 2020 showed elevated liver function tests, with an ALP of 300 IU/L and an ALT of 50 IU/L, which prompted a repeat CT scan in February 2021 (Figure 2). This CT scan report described diffuse ductal wall thickening with increased enhancement within the intrahepatic, hepatic, cystic, and common ducts. However, no significant extrahepatic biliary duct dilatation was seen. The gallbladder was not distended, which made its assessment difficult. These appearances were less conspicuous than in the previous CT scan from September 2020, which also exhibited mild common bile duct dilatation (10 mm), but they were new compared to a CT scan performed in January 2020. Due to the distribution of these findings and the lack of sepsis symptoms, this was reported as secondary to cholangiopathy. The radiologist checked the patient’s records, noticing abnormal liver function tests and after the literature review, considered secondary cholangitis due to pembrolizumab as the probable cause for these signs. Hepatology advice was to commence empirical steroids without biopsy, due to the impact of the COVID pandemic on diagnostic procedures.

## Outcome and follow-up

**Figure 3.**
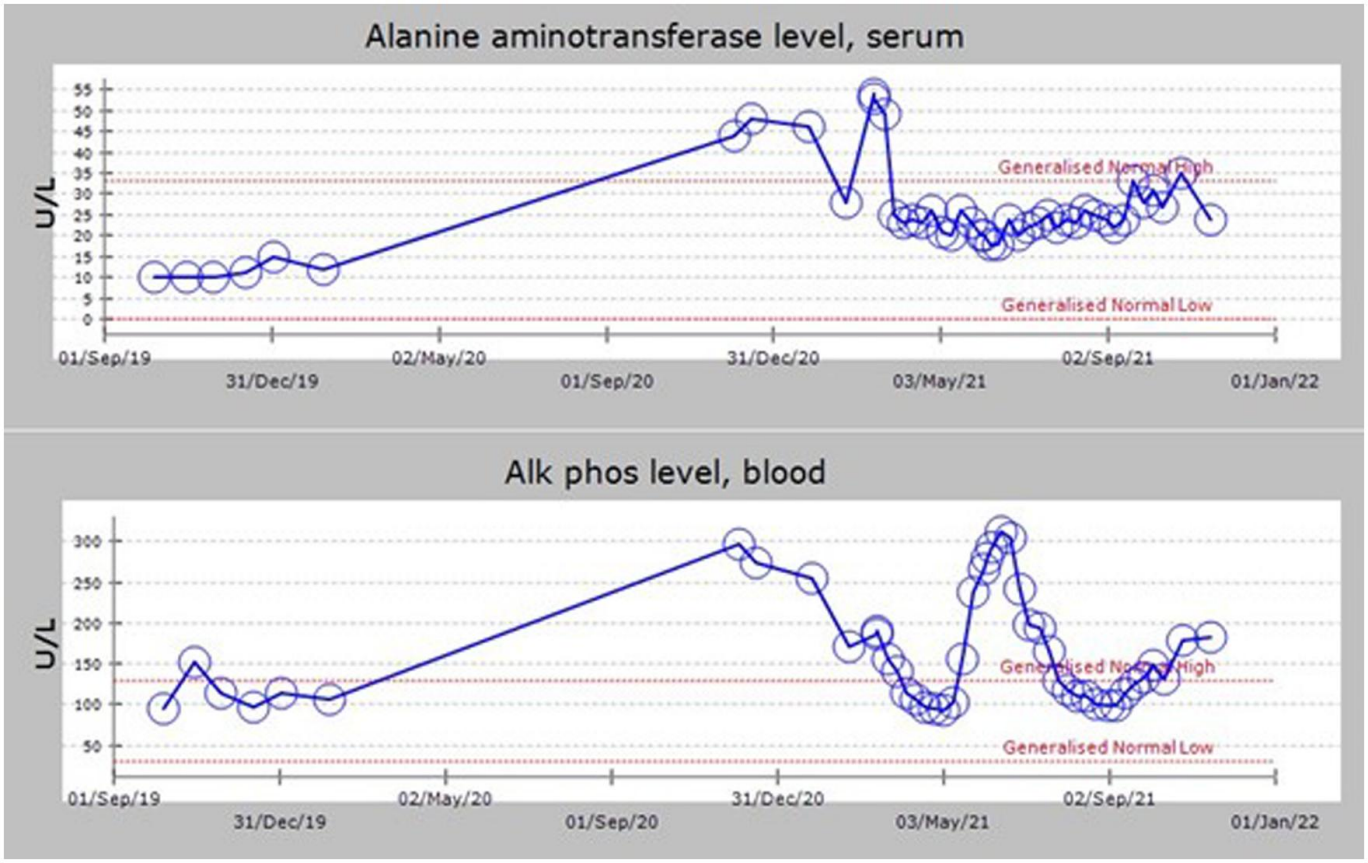
Liver function tests with time as the prednisolone dosage is changed.

Pembrolizumab succeeded in shrinking the NSCLC tumour despite evidence of autoimmune side effects. The patient responded well to prednisolone 20 mg once daily for the treatment of her cholangitis, with alanine aminotransferase levels falling to 30 IU/L and alkaline phosphatase falling to 100 IU/L. Reducing the prednisolone by 5 mg weekly led to the liver function tests returning to aberrant high values. Therefore, the 20 mg daily dosage was reinstated and then titrated down at a slower pace, leading to stabilized liver function tests in the normal range (Figure 3). Due to a further relapse in liver function, the prednisolone was yet again increased to 20 mg daily in September 2021, despite concerns about bone deterioration. In the interim, the ALT levels have remained normal, while ALP continues to rise. Further investigations revealed osteoporotic insufficiency fractures to be the cause of the ALP rise.

## Discussion

The CT scan report in September 2020 did not report any abnormalities in the bile ducts. In retrospect, there is clear evidence of cholangitis, which was diagnosed in the next follow-up CT and flagged up to the responsible clinician. Due to a combination of the ongoing COVID-19 pandemic and the patient’s reluctance to leave her house, no blood tests were performed for a year after the immunotherapy was finished. Without knowledge of the raised liver function tests, radiology did not have clinical suspicion to closely examine the bile ducts, and the changes were subtle.

Owen et al.^5^ have established that immune-related adverse events often start within 4 months of starting PD-1 therapies and that immune-related adverse events are different from previous autoimmune incidents, which this case study is consistent with. While Owen et al.’s study cites examples of delayed colitis, pneumonitis, and others, there is no record of delayed cholangitis. In general, checkpoint inhibitors can cause adverse toxic events in any organ, and recent data suggest 10%-17% of these can be serious or fatal.^6^ In 2022, Tanaka et al.^7^ reported a case of immune-related sclerosing cholangitis with a late onset 4 months after the discontinuation of pembrolizumab. Typically, case reports of pembrolizumab-induced sclerosing cholangitis present with raised ALP and ALT with normal IgG, such as the case reported by Tahboub Amawi et al.^8^ One sclerosing cholangitis case in the literature reports that the liver enzyme levels became so high that the patient met the criteria for discontinuation of pembrolizumab after 4 cycles.^9^ Other notable cases in the literature include the presentation of haemobilia alongside irreversible sclerosing cholangitis, which showed only a moderate response to corticosteroids after 5 cycles of pembrolizumab.^10^ A recent review of checkpoint inhibitor-induced immune-related cholangitis found that the median ALP value was 1328 IU/L and the medial ALT value was 156 IU/L, both substantially higher than the values recorded for this patient.^11^ This patient had a history of Graves’ disease and suspected autoimmune colitis from the immunotherapy, potentially putting her at risk for immune toxicity. Clinicians prescribing PD-L1 therapies should consider monitoring their patient’s liver function for a year following the end of their treatment.

## Learning points

- Cholangitis can appear up to a year after completing a course of Pembrolizumab treatment.
- Agoraphobia during the COVID-19 pandemic may have delayed the diagnosis.
- The diagnosis was missed on the first CT scan due to low clinical suspicion, but was accurately diagnosed and flagged up to the clinicians in the subsequent CT, which was reviewed by a GI radiologist.
